# *IGH* :: *IL3* ‐Rearranged B‐Cell Precursor Acute Lymphoblastic Leukemia With Hypereosinophilia in a Child With a Novel *PAX5* Germline Variant

**DOI:** 10.1002/gcc.70080

**Published:** 2025-09-22

**Authors:** Bartosz Urbański, Karolina Miarka‐Walczyk, Zuzanna Urbańska, Marta Wąsikowska, Elżbieta Sałacińska‐Łoś, Karolina Bukowska‐Strakova, Monika Lejman, Ewelina Jaroszek, Barbara Piątosa, Wojciech Młynarski, Agata Pastorczak, Szymon Janczar

**Affiliations:** ^1^ Department of Pediatrics, Oncology and Hematology Medical University of Lodz Lodz Poland; ^2^ Department of Genetic Predisposition to Cancer Medical University of Lodz Lodz Poland; ^3^ Department of Pediatric Surgery and Oncology Medical University of Lodz Lodz Poland; ^4^ Department of Clinical Immunology and Transplantology, Institute of Pediatrics Jagiellonian University Medical College Krakow Poland; ^5^ Laboratory of Genetic Diagnostics Medical University of Lublin Lublin Poland; ^6^ Histocompatibility Laboratory Children's Memorial Health Institute Warsaw Poland

**Keywords:** acute lymphoblastic leukemia, eosinophilia, germline mutation, JAK–STAT pathway, leukemia predisposition, optical genome mapping, *PAX5*

## Abstract

B‐cell precursor acute lymphoblastic leukemia (BCP‐ALL) with the t(5;14)(q31;q32) chromosomal translocation resulting in an *IGH*::*IL3* fusion is an exceptionally rare lymphoid malignancy presenting with hypereosinophilia. Germline *PAX5* alterations, including sequence variants and deletions, are associated with a selective susceptibility to BCP‐ALL and lymphomas, based on a limited number of affected families worldwide. Here, we report a 6‐year‐old male with BCP‐ALL with the t(5;14)(q31;q32) translocation and harboring a novel constitutional *PAX5* variant (NM_016734.3:c.[295dup];[=], p.[(Ile99Asnfs*3)];[(=)]). Despite the low representation of the leukemic clone in the bone marrow, the *IGH::IL3* fusion was identified by both fluorescent in situ hybridization (FISH) and optical genome mapping (OGM). Although hypereosinophilia poses a risk of multiorgan damage, our patient exhibited only pneumonia and asymptomatic neuroimaging alterations in the central nervous system. The patient achieved remission by the end of induction and is currently continuing maintenance therapy. In line with existing literature, familial segregation of the *PAX5* variant demonstrated high but incomplete penetrance, as two leukemia‐free mutation carriers displayed only subclinical B lymphocyte maturation abnormalities without hypogammaglobulinemia. Our report underscores the diagnostic utility of OGM, which unequivocally demonstrated the characteristic translocation. Typically, BCP‐ALL in affected family members exhibits secondary somatic aberrations involving the wild‐type *PAX5* allele, including structural variants or loss of heterozygosity (LOH) of chromosome 9p, as well as *PAX5* somatic mutations. To our knowledge, this is the first human report aligning with animal models, which suggests that secondary alterations activating the JAK–STAT pathway may potentially contribute to leukemogenesis in a *PAX5* mutation background.

## 1 Introduction

According to the fifth edition of the World Health Organization Classification of Hematolymphoid Tumors, the *IGH*::*IL3* fusion defines a distinct molecular subtype of B‐cell precursor acute lymphoblastic leukemia (BCP‐ALL) [1]. Despite this recognition, the t(5;14)(q31;q32) chromosomal translocation remains an extremely rare trigger of leukemia, with an estimated frequency of less than 1% of all BCP‐ALL cases [2]. This rearrangement leads to the juxtaposition of the *IGH* enhancer (situated on 14q32.1) to the *IL3* gene (located on 5q31.1), thereby promoting interleukin‐3 (IL‐3) expression in leukemic cells. The consequence of the IL‐3 overproduction is hypereosinophilia, a clinical hallmark of this entity [3].

The *PAX5* gene encodes a paired box domain transcription factor, which acts as a critical regulator of B cell progenitor differentiation from the early stages of maturation. Consistently, *PAX5* alterations (rearrangements, deletions, and sequence variants) that lead to the suppression or loss of its physiological function are involved in BCP‐ALL development in approximately 30% of patients [4]. While *PAX5* somatic lesions are extensively documented in pediatric B‐cell lymphoid malignancies, a selective predisposition to lymphoid neoplasia has recently been described in 28 patients from 14 families carrying germline *PAX5* alterations [5, 6, 7, 8, 9].

Here, we present a novel damaging constitutional variant of *PAX5* in a pediatric patient who developed *IGH*::*IL3*‐rearranged BCP‐ALL. This report suggests that somatic activation of the JAK–STAT signaling pathway may contribute to leukemogenesis in *PAX5* mutation carriers.

## 2 Case Report

**FIGURE 1:**
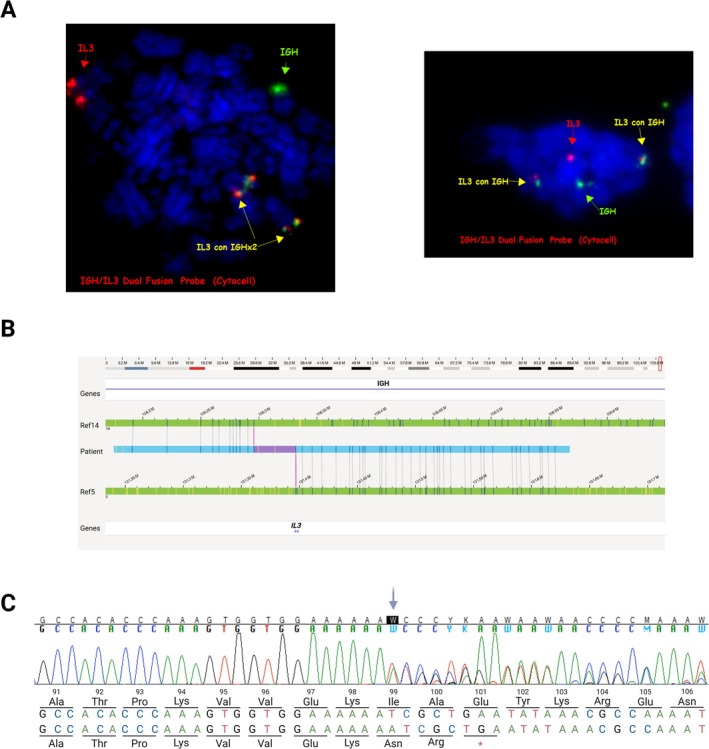
(A) FISH analysis demonstrating the presence of the *IGH::IL3* fusion in 2.67% of bone marrow smears (interphase nuclei) and 8.7% of bone marrow cultures (metaphases) (Cytocell Ltd., Oxford Gene Technology, Cambridge, United Kingdom). (B) Visualization of an interchromosomal translocation involving the *IL3* gene and the IGH region, detected by optical genome mapping. References for chromosome 14 (upper bar) and chromosome 5 (lower bar) are shown in green, while the patient's map is marked in blue. (C) Sanger sequencing chromatogram illustrating a pathogenic frameshift duplication in the *PAX5* gene (c.295dup, p.(I99Nfs*3)) with the stop codon highlighted.

A 6‐year‐old boy was admitted to the pediatric hematology and oncology unit due to general weakness, fever, cough, and limb pain persisting for 2 weeks, accompanied by laboratory abnormalities. A complete blood count revealed hyperleukocytosis (292 × 10^9^/L) with 78% eosinophils and 1% undifferentiated cells in the smear, thrombocytopenia (44 × 10^9^/L), and mild anemia (104 g/L). The child had no history of chronic illness. His grandfather was diagnosed with prostate cancer, and his aunt was treated for breast neoplasm. No hematological disorders or malignancies were reported in the family. Bone marrow (BM) cytology revealed the presence of approximately 70% of cells of the eosinophilic lineage, predominantly segmented eosinophils, and 13% of blasts morphologically suggestive of the myeloid line (Supporting Information S1). Given the deteriorating condition of the patient and the significant risk of leukostasis and organ damage, cytoreductive therapy (cytarabine, hydroxyurea) was initiated according to the AML BFM 2019 Protocol. The following day, BM flow cytometry results were obtained. While 89% of the cells were derived from the eosinophilic lineage, leukemic blasts constituted only 4% of all nucleated cells and exhibited an abnormal immunophenotype characteristic of B‐lineage lymphoblasts (hyperexpression of CD10, CD58, CD123, and CD66c, alongside low expression of CD38 and CD81; Supporting Information S2). The patient was eventually diagnosed with BCP‐ALL and treated according to the AIEOP BFM ALL 2017 Protocol. In addition, fluorescent in situ hybridization (FISH) revealed the presence of the *IGH*::*IL3* rearrangement in 2 out of 23 analyzed metaphases (Figure 1A). Importantly, this clonal marker was absent in eosinophils. The t(5;14)(q31;q32) chromosomal translocation, resulting in the *IGH*::*IL3* fusion, was also confirmed using optical genome mapping (OGM) (Figure 1B).

**FIGURE 2:**
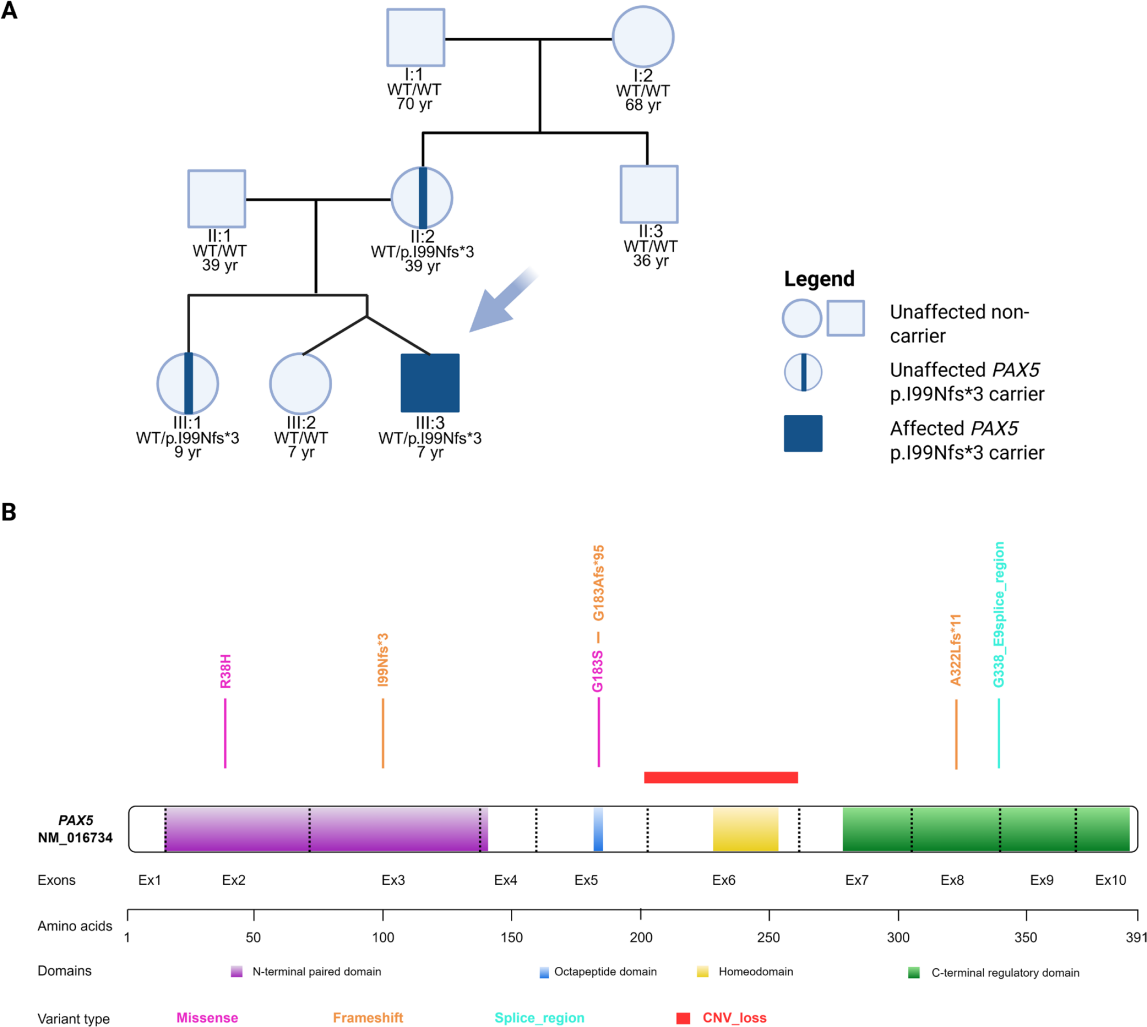
(A) Pedigree analysis of the *PAX5*:c.295dup, p.(I99Nfs*3). In addition to the proband (III:3), the germline variant in *PAX5* was identified in the asymptomatic mother (II:2) and non‐twin sister (III:1). (B) Schematic representation of *PAX5*, showing its domains and the germline variants reported to date (detailed characterization of the variants is provided in Supporting Information S6). WT—wild type.

No further pathogenic chromosomal aberrations were identified using karyotyping and OGM. However, in diagnostic RNA sequencing, we detected a likely pathogenic (according to the ACMG Guidelines: PVS1 Very Strong and PM2 Moderate) frameshift duplication in the *PAX5* gene (NM_016734.3:c.[295dup];[=], p.[(Ile99Asnfs*3)];[(=)]) exhibiting a variant allele frequency (VAF) of 49%. A single‐base duplication of adenine occurring immediately after a stretch of six consecutive adenines results in the formation of a stop codon and premature termination of the protein chain (Figure 1C, Supporting Information S3). The MutationTaster tool predicts that this mutation triggers nonsense‐mediated decay (NMD). This novel *PAX5* variant has not been previously reported in the Franklin by Genoox, gnomAD, VarSome, or Pecan St. Jude Cloud databases (as of August 2025). Due to the discrepancy between the representation of blasts in the bone marrow and the *PAX5* VAF in molecular testing, buccal swabs were collected, ultimately confirming the presence of a heterozygous constitutional mutation. No additional pathogenic variants predisposing to cancer were identified by whole‐exome sequencing. Familial segregation analysis revealed that the patient's healthy mother and sister were carriers of the deleterious alteration, which occurred de novo in the proband's mother (Figure 2A).

Initial clinical imaging unveiled moderate inflammatory changes in the lungs accompanied by a small amount of pleural effusion. The brain MRI demonstrated numerous diffuse foci of elevated signal intensity, potentially corresponding to leukemic infiltrates. However, no abnormal cells were identified in the cerebrospinal fluid, and the child presented no neurological symptoms. We tentatively assumed the abnormal neuroimaging result to be eosinophilia‐related. Conversely, no cardiac compromise was observed, as previously described in several patients with *IGH*::*IL3*‐rearranged BCP‐ALL, and the extent of organ damage was mild compared to previous reports [2, 3, 10].

The boy exhibited a relatively rapid decrease in leukocyte count during induction (prednisone, vincristine, and daunorubicin), which fell below 10 × 10^9^/L by the 11th day of therapy. On the 15th day of treatment, BM cytometric analysis revealed 1.27% lymphoblasts with a phenotype corresponding to the diagnosis, while eosinophilic forms were sparse. By the 33rd day of therapy (end of induction), the patient achieved complete cytological and cytometric remission. Due to the low percentage of blasts at diagnosis, it was not possible to assess minimal residual disease (MRD) using molecular methods. The patient was ultimately classified into the medium‐risk group according to the AIEOP BFM ALL 2017 Protocol guidelines. In the follow‐up brain MRI, the resolution of the initially described changes was observed. No significant organ damage or toxicity related to the diagnosis and therapy was noted, apart from *Pseudomonas aeruginosa* septicemia and transient severe hypertension during re‐induction. Currently, the patient remains in remission and is receiving oral maintenance therapy.

To assess the potential impact of the *PAX5* variant on B cell maturation, we conducted a cytometric analysis of lymphocytes from the peripheral blood of unaffected mutation carriers (the patient's mother and sister). A detailed immunophenotypic characterization demonstrated subclinical abnormalities in B cell maturation without concomitant hypogammaglobulinemia (Supporting Information S4). All investigations described above were performed in certified national reference laboratories (critical parameters of the assays are provided in Supporting Information S5).

## 3 Discussion

BCP‐ALL with the t(5;14)(q31;q32) chromosomal translocation resulting in the *IGH*::*IL3* fusion represents a fascinating example of a rare leukemia subtype characterized by a specific paraneoplastic syndrome. Excessive eosinophilia frequently manifests as multi‐organ damage, including cardiomyopathy, thromboembolic events (particularly within the central nervous system), pulmonary infiltrates, organomegaly, and skin involvement [3, 10]. Our patient initially exhibited only pneumonia and asymptomatic neuroimaging changes in the central nervous system. Despite its distinct clinical presentation, *IGH*::*IL3*‐rearranged leukemia poses numerous diagnostic challenges, primarily due to the low percentage of blasts in the bone marrow and the necessity to exclude much more common reactive hypereosinophilic syndromes. Moreover, detecting characteristic rearrangement using classical cytogenetic or molecular methods is often difficult, as *IGH* fusions are not satisfactorily registered in short‐read RNA sequencing [11]. Our report advocates for the application of OGM, which unequivocally demonstrated the translocation. Several difficulties are still faced in the management of this subtype of leukemia, particularly concerning MRD monitoring with molecular methods. The published case series encompass diverse populations, age groups, and treatment protocols, making it challenging to draw meaningful prognostic conclusions [2, 3, 10]. There is a strong need to explore new relapse markers beyond the increase in eosinophil counts.

Based on large genomic research on childhood BCP‐ALL, somatic *PAX5* alterations are among the most frequent, registered in approximately 30% of cases [4]. Conversely, germline *PAX5* variants linked to leukemia susceptibility syndrome have been reported in isolated familial cases (Figure 2B, Supporting Information S6). Most of them are missense hypomorphic mutations resulting in decreased PAX5 activity. Recently, Bettini et al. reported a novel frameshift mutation (*PAX5:* c.548delG, p.(Gly183Alafs*84)) occurring within a highly conserved octapeptide domain, resulting in premature termination of the protein chain [6]. In contrast, the variant identified in our patient is located in the N‐terminal paired domain characteristic of the Pax family. This region is critical for recognizing and binding DNA sequences, the transcription of which is subsequently modulated by the C‐terminal regulatory domain [12]. To date, only two families carrying the germline missense mutation (c.113G>A, p.(Arg38His)) in this region have been reported [7, 9]. Although reliable estimation of the penetrance of damaging *PAX5* variants requires further studies, most families displayed high (~30% or more) but incomplete penetrance [6, 7, 8, 9, 13, 14, 15, 16, 17]. Consistently, reduced penetrance was observed in the proband's family, in which two asymptomatic mutation carriers displayed only subclinical B lymphocyte maturation abnormalities. Although our patient has a dizygotic twin, the *PAX5* variant was identified in the non‐twin sister. Flow cytometry analysis of B cells revealed a decreased proportion of immature (CD19+ CD21low), nonswitched (CD27+ IgD+/CD19+), and IgG memory (IgG+ IgM−/CD19+) B lymphocytes in both carrier family members (Supporting Information S4). In contrast, previous studies involving patients with *PAX5* variants restricted to the octapeptide domain reported a reduction predominantly in memory B cells (IgD‐ CD27+) [6, 14]. Our findings may suggest that mutations in the N‐terminal paired domain similarly disrupt B cell development, but delineating a distinct pattern of these abnormalities remains challenging.

To better characterize the impact of specific variants on protein function and their association with phenotypes, further clinical observations, as well as animal models and functional studies, are needed. Extending clinical knowledge remains challenging due to the limited number of reported families and the relatively short follow‐up duration. Drawing from in vivo animal studies, it may be speculated that patients with *PAX5* germline mutations likely develop a latent preleukemic clone, with only a subset experiencing a secondary event that drives malignant transformation [13]. In recent years, some research has suggested that exposure to infectious agents in early childhood may serve as a key trigger for this progression [18]. Further, Escudero et al. demonstrated that mice genetically predisposed to BCP‐ALL, including those with constitutional *PAX5* heterozygosity, exhibited a distinct gut microbiota profile. Disruption to this profile during early life, induced solely by antibiotic treatment, was sufficient to initiate leukemogenesis, even in the absence of an infectious agent. The authors hypothesized that gut microbiota profiling could serve as an early biomarker for malignant transformation and that microbiome modulation may represent a promising preventive strategy [19]. The infectious hypothesis aligns with the previously proposed pivotal role of activation‐induced cytidine deaminase (AID) in the generation of secondary genetic alterations. Notably, AID expression in precursor B lymphocytes is upregulated in response to inflammatory stimuli triggered by a restricted subset of microorganisms [20]. Further research is necessary to validate these findings in human clinical settings, but the interplay with early‐childhood infectious exposure may be proposed as one of the factors explaining incomplete cancer penetrance.

To date, the development of BCP‐ALL in the vast majority of cases with *PAX5* germline mutations has been attributed to second‐hit somatic variants affecting the wild‐type allele (structural variants, loss of heterozygosity (LOH) on chromosome 9p, and *PAX5* point mutations) [6, 7, 8]. However, it has been demonstrated that secondary alterations involving other signaling pathways, such as JAK–STAT or RAS, may also lead to leukemia development in germline *PAX5* mutation carriers. Heltemes‐Harris et al. showed that all transgenic mice with *PAX5* haploinsufficiency and activated Stat5 ultimately developed BCP‐ALL [21]. In another study, it was demonstrated that the pressure exerted by infectious agents in patients with cancer predisposition syndromes may lead to the accumulation of secondary *Jak3* alterations. Inhibition of the JAK–STAT pathway has emerged as a promising therapeutic strategy, as it was shown in murine models to induce leukemic cells apoptosis in animals harboring *PAX5* mutations [18]. Furthermore, Casado‐García et al. [22] reported in a mouse model that the early‐life administration of ruxolitinib, a JAK 1/2 inhibitor, may even prevent carriers of *PAX5* germline variants from developing leukemia. Simultaneously, based on animal models, the hyperexpression of CD123, an IL‐3 receptor, on *IGH*::*IL3*(+) leukemia cells results not only in their autocrine stimulation but also leads to the Stat5 overactivation [23]. These observations provide a rationale for the potential JAK–STAT‐dependent expansion of the leukemic clone in our patient and are consistent with the previously proposed infectious hypothesis of leukemia development in individuals with cancer predisposition syndromes. To our knowledge, this is the first description of BCP‐ALL development in *PAX5* germline leukemia predisposition syndrome, resulting from secondary alterations that may potentially activate the JAK–STAT pathway. Unfortunately, we could not use bulk RNA sequencing data to explore this hypothesis due to the low percentage of blasts at ALL diagnosis. Further studies, including single‐cell RNA sequencing, could potentially facilitate the understanding of the mechanisms contributing to leukemogenesis in this inherited condition. Beyond these molecular aspects, extending knowledge about cancer penetrance and long‐term outcomes in *PAX5* mutation carriers may enable the development of effective surveillance strategies for this leukemia predisposing syndrome.

## 4 Conclusions

*IGH::IL3* BCP‐ALL represents a distinct subtype of leukemia associated with significant challenges in diagnosis, treatment, and long‐term management. OGM is emerging as a promising diagnostic option, enabling the detection of characteristic chromosomal translocations with high sensitivity. This first reported case of *IGH::IL3* BCP‐ALL developing on the background of *PAX5*‐related cancer predisposition syndrome provides novel insights into leukemogenesis and suggests a potential role for activation of the JAK–STAT pathway. Further functional studies and longitudinal clinical follow‐up are essential to elucidate the variable penetrance of specific *PAX5* variants.

## Author Contributions

B.U. gathered data, conducted a formal analysis, and wrote the manuscript. K.M.W. and Z.U. performed the molecular research and ensured its visualization. M.W. gathered clinical data. E.S.Ł. was responsible for the bone marrow cytological examination and provided its visualization. K.B.S. performed flow cytometry for the diagnosis and provided dot plots. E.J. and B.P. conducted flow cytometry of B cells and provided dot plots. M.L. was responsible for FISH and its visualization. W.M. and A.P. critically reviewed the original draft and supervised the work. S.J. formulated the research idea, critically reviewed the original draft, and supervised the work. All authors have read and agreed to the published version of the manuscript.

## Ethics Statement

The work described in this article has been carried out following The Code of Ethics of the World Medical Association (Declaration of Helsinki) for experiments involving humans; EU Directive 2010/63/EU for animal experiments; and uniform requirements for manuscripts submitted to biomedical journals. This study was approved by the Local Ethical Committee at the Medical University of Lodz (decision No. RNN/213/22/KE dated September 13, 2022).

## Consent

The patient's guardian has provided informed consent to publish the case report.

## Conflicts of Interest

The authors declare no conflicts of interest.

## Supporting information


**Data S1:** Supporting Information.

## Data Availability

The data that support the findings of this study are available from the corresponding author upon reasonable request.
