# Supplementary material for: IGH :: IL3 ‐Rearranged B‐Cell Precursor Acute Lymphoblastic Leukemia With Hypereosinophilia in a Child With a Novel PAX5 Germline Variant
Source: Genes Chromosomes Cancer. 2025 Sep 22;64(9):e70080. doi: 10.1002/gcc.70080 (PMC12452213; doi:10.1002/gcc.70080)
Supplement: Supplementary file 1 — Data S1: Supporting Information. [file GCC-64-e70080-s001.docx]

**Supporting Information**

**S1 Cytological examination of the bone marrow aspirate at diagnosis revealing the presence of 13% blasts and a significant number of eosinophilic lineage cells (May-Grünwald-Giemsa, 600x and 1000x)**

**
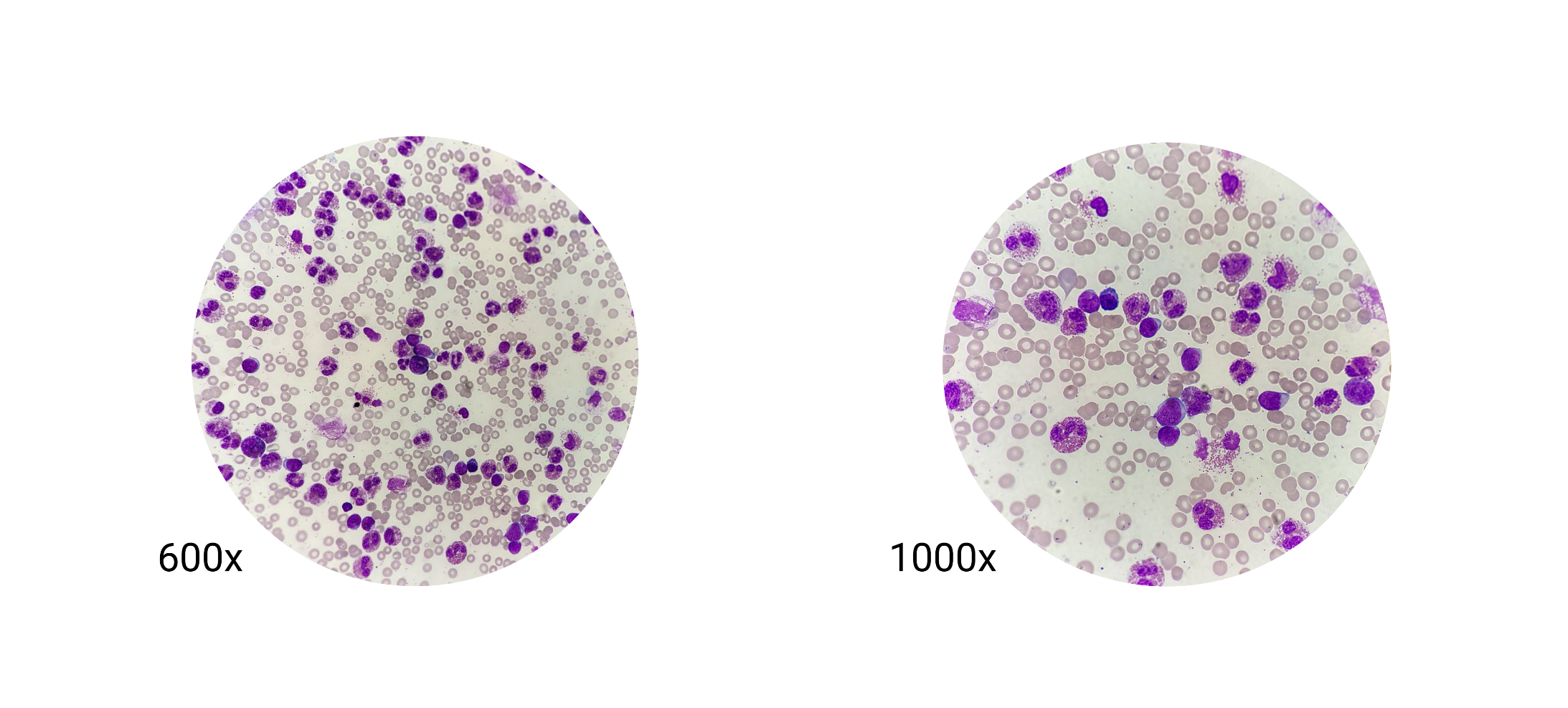
**

**S2 Immunophenotyping of bone marrow at diagnosis using flow cytometry**


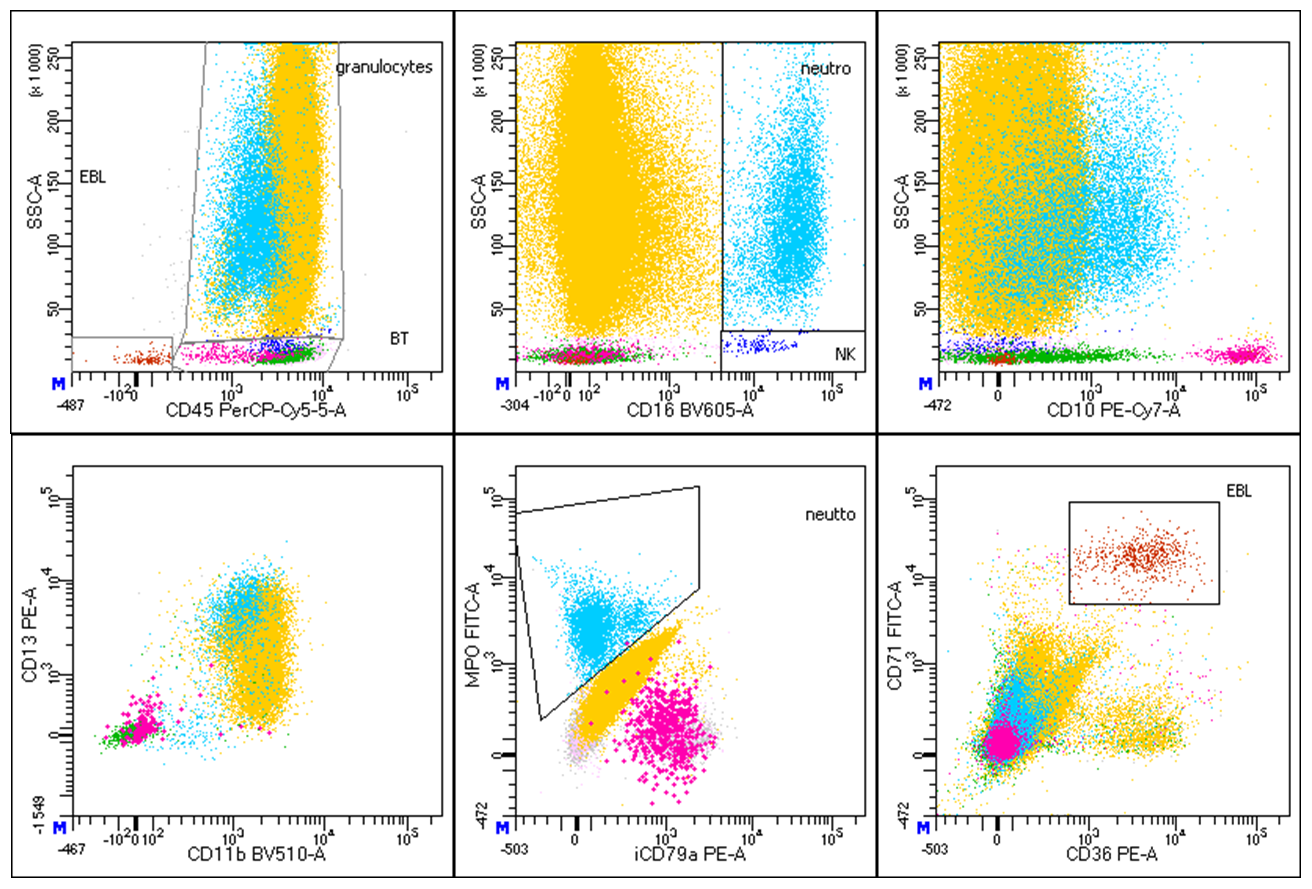


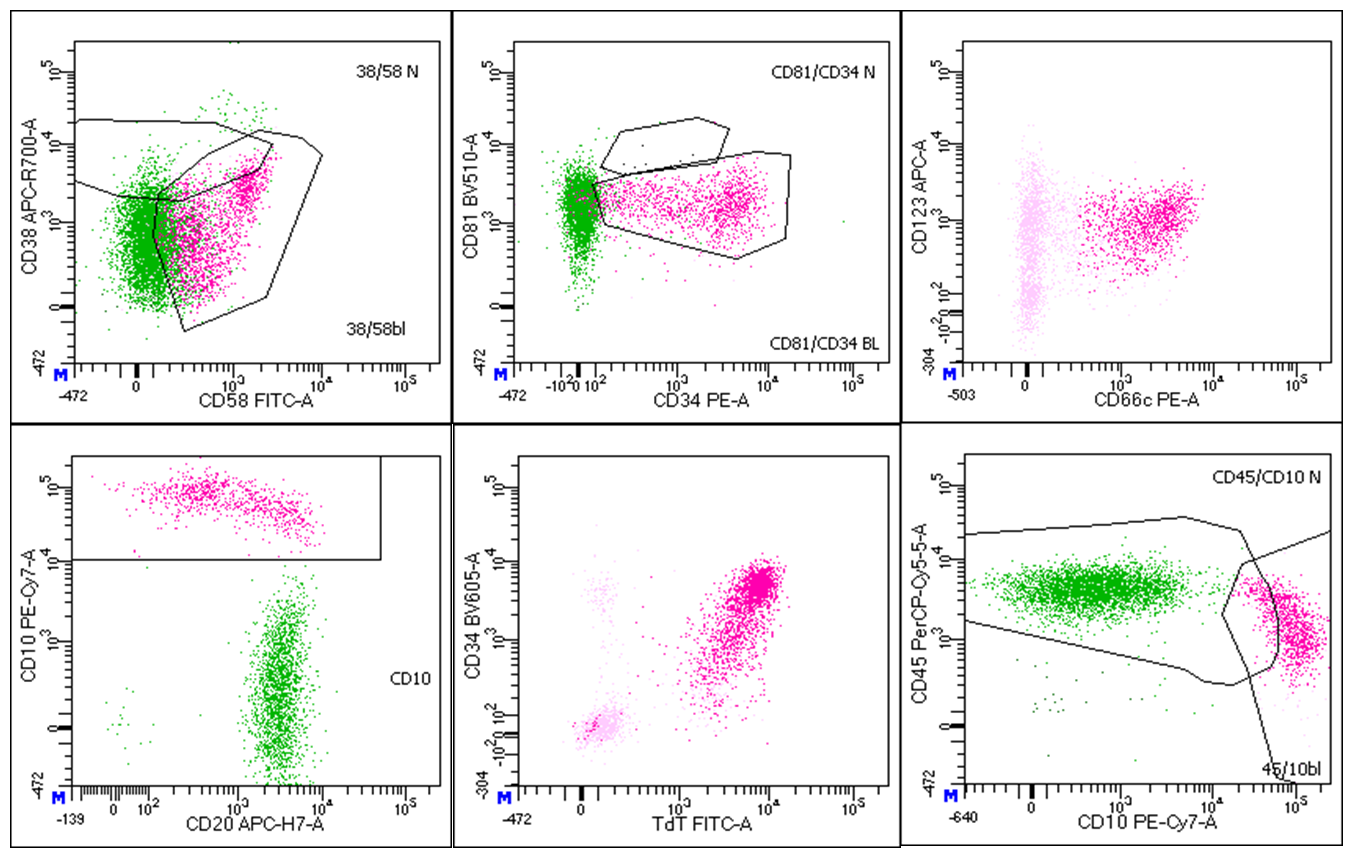


| CD45 | medium | CD10 | bright (hyperexpression) | CD1a | negative | MPO | dim |
| --- | --- | --- | --- | --- | --- | --- | --- |
| CD34 | heterogeneous 78% | **CD19** | bright | **CD2** | negative | **iLysozyme** | negative |
| CD117 | negative | **CD20** | partially positive 1 40% | **CD3** | negative | **CD11b** | negative |
| CD133 | negative | **CD22** | medium | **iCD3** | negative | **CD11c** | negative |
| HLA-DR | bright | **iCD22** | medium | **CD4** | negative | **CD13** | negative |
| iTdT | bright | **CD24** | no data | **CD5** | negative | **CD14** | negative |
| CD11a | dim | **iCD79a** | bright | **CD7** | negative | **CD15** | negative |
| CD38 | medium | **kappa** | not done | **CD8** | negative | **CD16** | negative |
| CD58 | bright | **lambda** | not done | **CD56** | negative | **CD33** | negative |
| CD73 | dim | **sIgM** | not done | **CD99** | bright | **CD64** | negative |
| CD81 | medium | **iIgM** | not done |  |  | **CD65w** | negative |
| CD36 | negative |  |  |  |  | **CD66c** | partially positive 2 51% |
| CD71 | negative |  |  |  |  | **CD123** | bright |
| CD235a | negative |  |  |  |  | **CD371** | negative |
| CD45RA | medium |  |  |  |  | **NG2** | negative |
|  |  |  |  |  |  | **CD41a** | negative |
|  |  |  |  |  |  | **CD42b** | negative |
|  |  |  |  |  |  | **CD61** | negative |

Abbreviations related to the expression of specific antigens:

- negative – below 10% of positive cells;
- weak positive – 10-50% of positive cells: - dim: the entire population shifted towards the positive cells, - partially positive 1: two distinct populations observed;
- strong positive – above 50% of positive cells: - medium: the entire population shifted towards the positive cells, - partially positive 2: two distinct populations observed, - bright: the entire population strongly positive, not overlapping with the negative cells, - heterogeneous – heterogeneous expression extending beyond 1.5 logs;
- i – intracellular expression.

**Standardized recording according to ‘Flow Diagnostic Essential’ Code:**

FDE:0.5%; STRONG:CD10,19,22,i22,34,38,45,45RA,68,66c,i79a,81,99,123,HLA-DR,iTdT; WEAK:CD11a,20,73,MPO; NEGATIVE:CD1a,2,3,i3,4,5,7,8,11b,11c,13,14,15,16,33,36,41a,42b,56,61,64,65w,71,117,133,235a,371,iLysozyme,NG2; BCP-ALL II/III with low percentage of blasts

**Conclusions:** The population of pathological cells (constituting approximately 0.5% of the nucleated cells) corresponds to the acute lymphoblastic leukemia (BCP-ALL) subtype II/III (staining for heavy chains was not performed). The hyperexpression of CD10, high expression of CD58, CD123, and CD66c, along with low expression of CD38 and CD81, indicates the pathological nature of these cells. Furthermore, eosinophilic cells dominate, constituting 89% of the nucleated cells, which may suggest B-lymphoblastic leukemia/lymphoma with *IGH*::*IL3* fusion subtype.

**S3A Sanger sequencing chromatogram illustrating bidirectional sequencing reads of the *PAX5* region harboring the mutation in a healthy individual (father) and a mutation carrier (proband)**

**
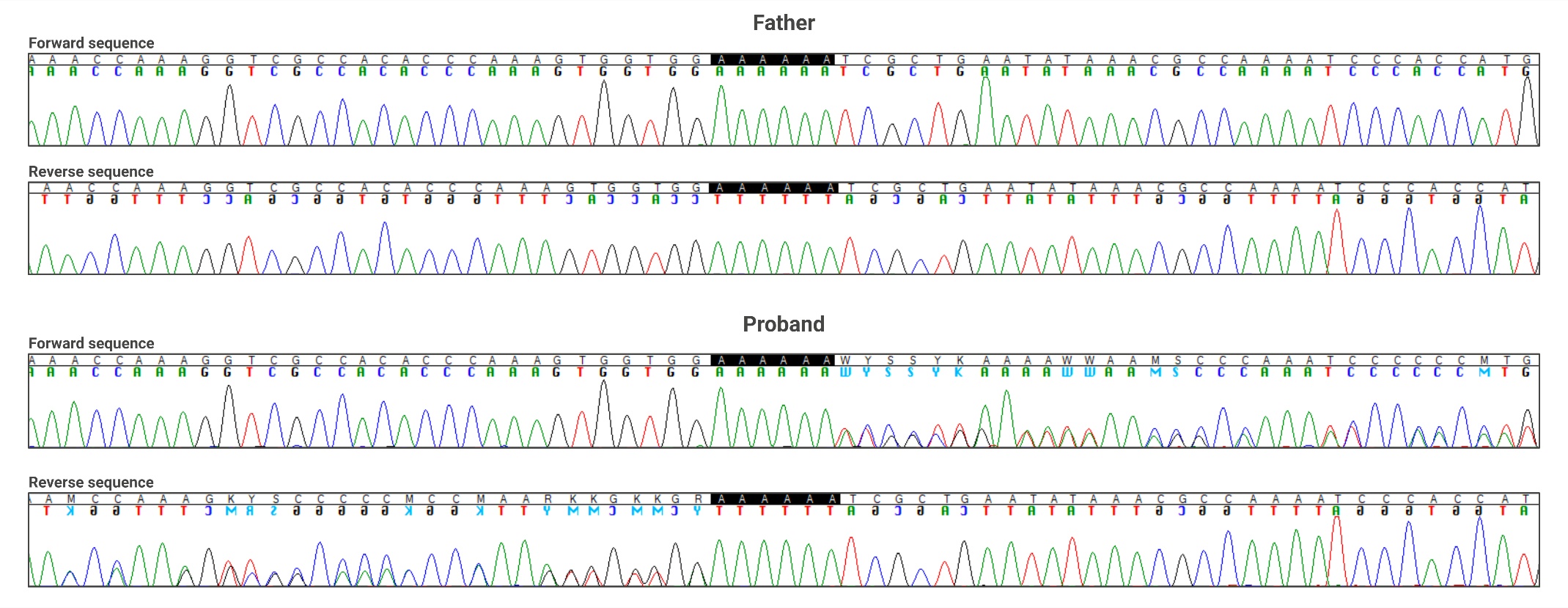
**

**S3B Integrated Genomics Viewer image displaying the described *PAX5* variant in whole-exome sequencing**

**
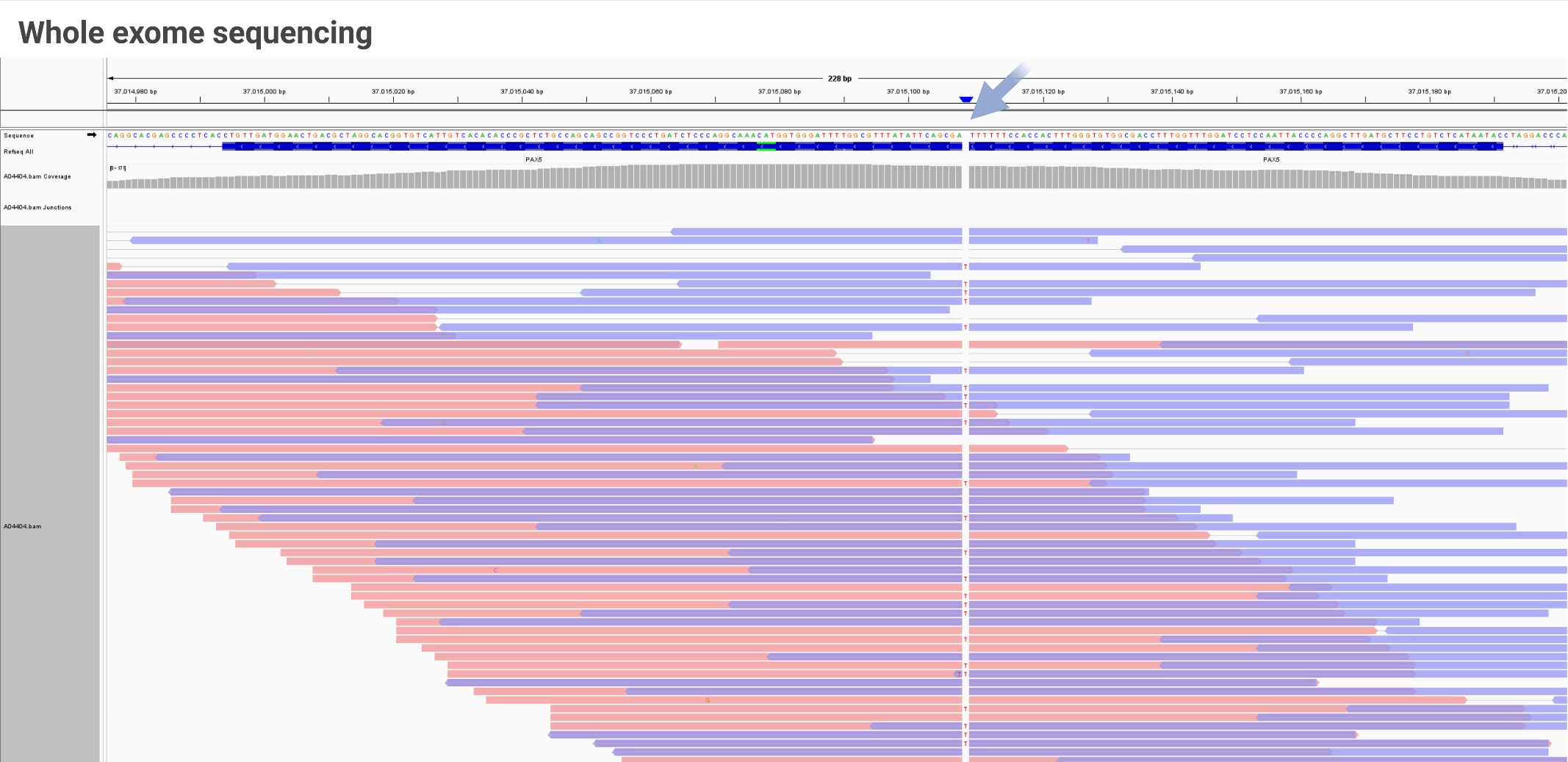
**

**S4A Detailed results of flow cytometry analysis of B lymphocytes from the peripheral blood of the proband’s mother (carrier of the *PAX5* germline variant)**

| B-lymphocyte subpopulation | Cytometric characterization | Percentage [%] | Reference value range |
| --- | --- | --- | --- |
| transitional | CD38+IgM++/CD19+ | 1.40 | 0.90 – 5.70 |
| immature | CD19+CD21low/lymphocytes | 0.23 | 0.40 – 2.00 |
| mature | CD27-/CD19+ | 76.66 | 53.50 – 86.70 |
| mature naïve | IgD+CD27-/CD19+ | 74.18 | 48.40 – 79.70 |
| memory cells | CD27+/CD19+ | 23.34 | 17.50 – 46.50 |
| ‘non-switched’ memory | CD27+IgD+/CD19+ | 3.69 | 7.00 – 23.80 |
| ‘switched’ memory | CD27+IgD-/CD19+ | 19.50 | 8.30 – 27.80 |
| IgM-only memory | IgD-IgM+/CD19+IgD-CD27+ | 12.30 | 2.50 – 30.50 |
| IgM-only memory | IgD-IgM+CD27+/CD19+ | 2.40 | 0.40 – 3.70 |
| activated | CD38lowCD21low/CD19+ | 2.00 | 1.60 – 10.00 |
| plasmablasts | CD38+++IgM-/CD19+ | 0.67 | 0.40 – 2.40 |
| typical IgG memory | IgG+IgM-CD27+/CD19+ | 0.03 | 0.70 – 9.20 |
| atypical IgG memory | IgG+IgM-CD27-/CD19+ | 0.04 | 0.50 – 4.30 |
| typical IgA memory | IgA+IgM-CD27+/CD19+ | 7.05 | 3.00 – 8.20 |
| atypical IgA memory | IgA+IgM-CD27-/CD19+ | 0.69 | 0.70 – 3.60 |
| germinal center cells | CD27+CD38+IgD-/CD19+ | 0.44 | 0.60 – 3.40 |
| centroblasts | CD27+CD38+IgD-CD184+/CD19+ | 0.25 | 0.20 – 2.10 |
| centrocytes | CD27+CD38+IgD-CD184-/CD19+ | 0.19 | 0.30 – 2.40 |
| plasma cells | CD138+/lymph | 0.13 | 0.03 – 1.00 |


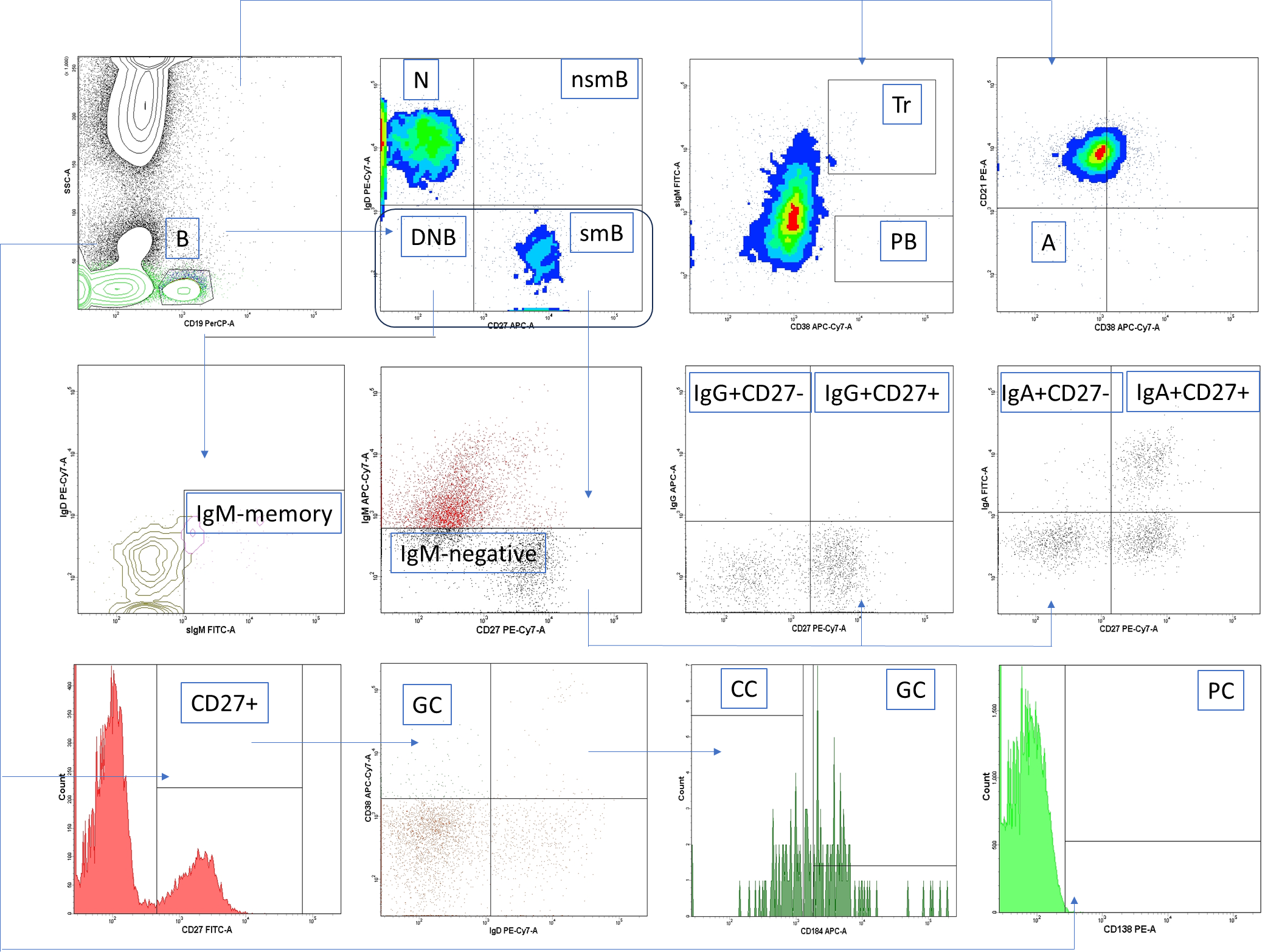


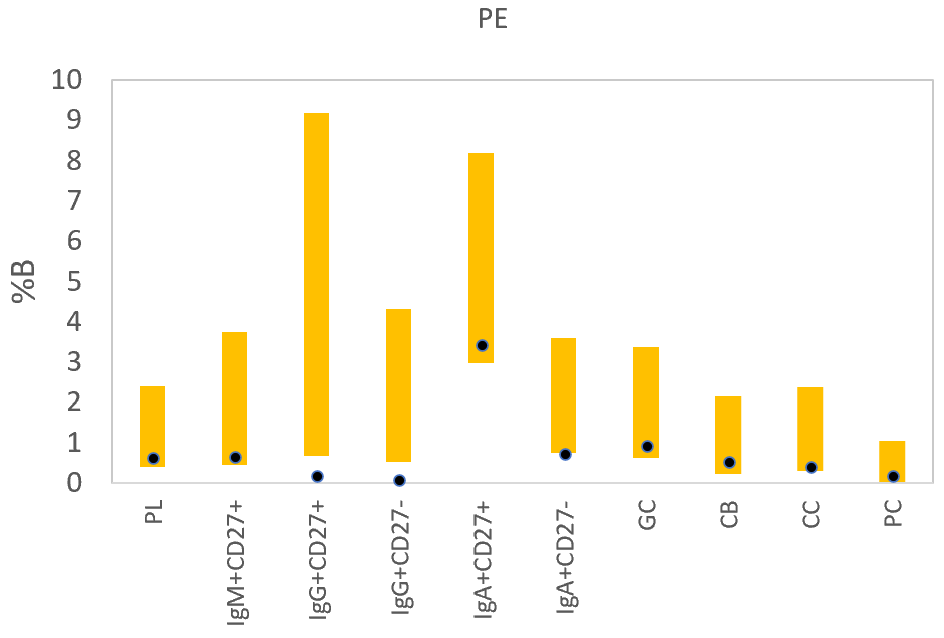

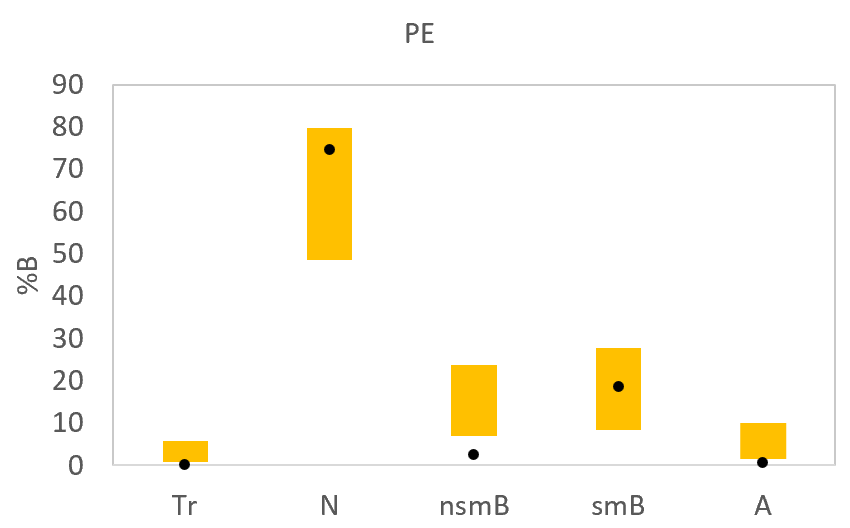


Abbreviations: B – B lymphocytes, N - mature naïve, nsmB – ‘non-switched’ memory, smB – ‘switched’ memory, DNB – double negative IgD-CD27-, Tr – transitional, PB – plasmablasts, A – activated, GC – germinal center cells, CC – centrocytes, CB – centroblasts, PC – plasma cells

**Conclusions:** Reduced percentage of immature B lymphocytes and ‘non-switched memory’ B cells. Decreased proportion of typical and atypical IgG memory B lymphocytes. Reduced percentage of germinal center cells, particularly centrocytes.

The reference value ranges are age-adjusted and based on data from a representative healthy population (1).

**S4B Detailed results of flow cytometry analysis of B lymphocytes from the peripheral blood of the eight-year-old proband’s sister (carrier of the *PAX5* germline variant)**

| B-lymphocyte subpopulation | Cytometric characterization | Percentage [%] | Reference value range |
| --- | --- | --- | --- |
| transitional | CD38+IgM++/CD19+ | 1.77 | 4.60 – 8.30 |
| immature | CD19+CD21low/lymphocytes | 0.35 | 0.80 – 4.10 |
| mature | CD27-/CD19+ | 79.48 | 53.30 – 81.40 |
| mature naïve | IgD+CD27-/CD19+ | 76.48 | 47.30 – 77.00 |
| memory cells | CD27+/CD19+ | 20.52 | 18.60 – 46.70 |
| ‘non-switched’ memory | CD27+IgD+/CD19+ | 4.47 | 5.20 – 20.40 |
| ‘switched’ memory | CD27+IgD-/CD19+ | 15.46 | 10.90 – 30.40 |
| IgM-only memory | IgD-IgM+/CD19+IgD-CD27+ | 4.07 | 7.00 – 23.40 |
| IgM-only memory | IgD-IgM+CD27+/CD19+ | 0.63 | 0.50 – 3.30 |
| activated | CD38lowCD21low/CD19+ | 1.69 | 2.30 – 10.00 |
| plasmablasts | CD38+++IgM-/CD19+ | 0.61 | 0.60 – 5.30 |
| typical IgG memory | IgG+IgM-CD27+/CD19+ | 0.16 | 0.20 – 8.10 |
| atypical IgG memory | IgG+IgM-CD27-/CD19+ | 0.05 | 0.20 – 5.00 |
| typical IgA memory | IgA+IgM-CD27+/CD19+ | 3.39 | 1.80 – 7.00 |
| atypical IgA memory | IgA+IgM-CD27-/CD19+ | 0.69 | 0.60 – 4.30 |
| germinal center cells | CD27+CD38+IgD-/CD19+ | 0.89 | 0.70 – 8.10 |
| centroblasts | CD27+CD38+IgD-CD184+/CD19+ | 0.51 | 0.30 – 4.20 |
| centrocytes | CD27+CD38+IgD-CD184-/CD19+ | 0.38 | 0.30 – 3.80 |
| plasma cells | CD138+/lymph | 0.16 | 0.10 – 1.10 |


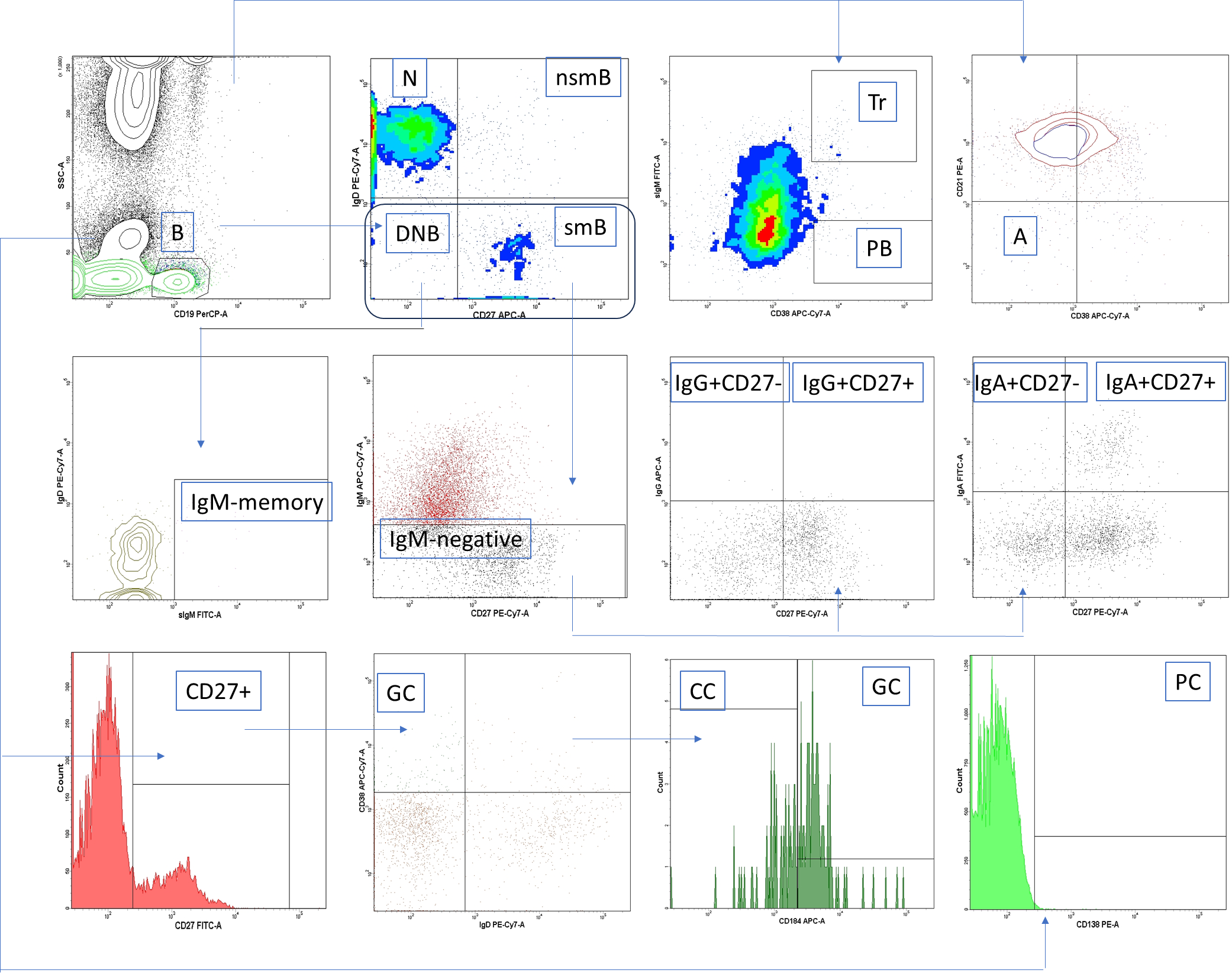


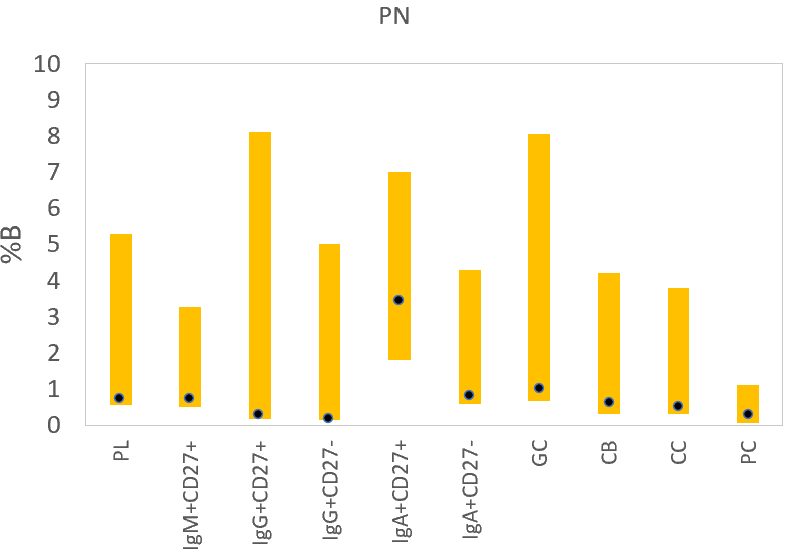

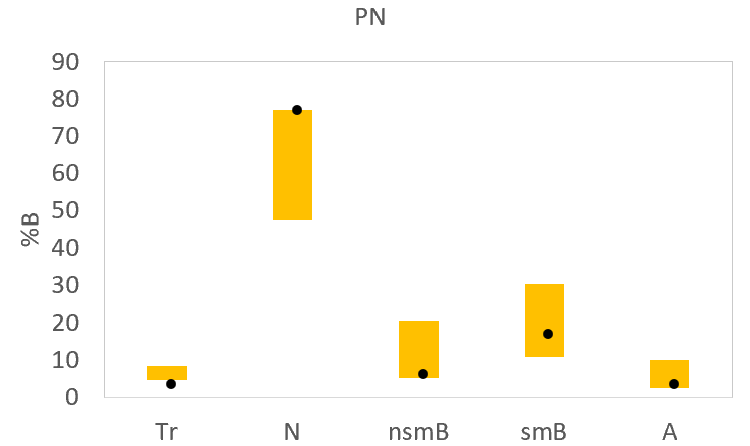


Abbreviations: B – B lymphocytes, N - mature naïve, nsmB – ‘non-switched’ memory, smB – ‘switched’ memory, DNB – double negative IgD-CD27-, Tr – transitional, PB – plasmablasts, A – activated, GC – germinal center cells, CC – centrocytes, CB – centroblasts, PC – plasma cells

**Conclusions:** Reduced percentage of transitional B lymphocytes, immature B lymphocytes, and non-switched memory B lymphocytes. Decreased proportion of IgM memory B lymphocytes within the memory B cell population. Lowered percentage of atypical IgG memory B lymphocytes. Reduced proportion of activated B lymphocytes.

The reference value ranges are age-adjusted and based on data from a representative healthy population (1).

**S5 Methods – critical parameters of the assays**

**A Karyotyping and fluorescent in situ hybridization (FISH)**

According to the AIEOP BFM ALL 2017 Protocol, GTG band staining and fluorescence in situ hybridization (FISH) tests were performed using molecular probes: Vysis LSI BCR/ABL Dual Color, Dual Fusion Translocation Probe Kit (Vysis, Abbot Molecular, Illinois, USA), XL KMT2A BA Break Apart Probe (MetaSystems Probes), XL t(12;21) ETV6/RUNX1 DF Translocation/Dual Fusion Probe (MetaSystems Probes), and E2A (TCF3) Breakapart (CytoCell, Cytocell Ltd., Oxford Gene Technology, Cambridge, United Kingdom) at the time of diagnosis. The arrangement of the probes' signals indicated a lack of chromosomal aberrations. The genetic examinations included an additional FISH test of material collected at the time of diagnosis, which showed the presence of the *IGH*::*IL3* (CytoCell, Cytocell Ltd., Oxford Gene Technology, Cambridge, United Kingdom) fusion in 2.67% of bone marrow smears (interphase nuclei) and 8.7% of bone marrow cultures (metaphases).

46,XY[30].ish t(5;14)(IL3+,IGH+;IGH+,IL3+)[2/23].nuc ish (IL3,IGH)x3(IL3 con IGH)x2[11/411], (ABL1,BCR)x2[200],(KMT2A)x2[200],(ETV6,RUNX1)x2[200],(TCF3)x2[200] *

*according to the ISCN 2024

**B RNA sequencing**

RNA sequencing was performed using the TruSight RNA Pan-Cancer panel (Illumina, San Diego, CA), which contains 1,385 cancer genes and enables fusion calling and variant detection within the panel. Twenty nanograms of RNA were processed according to the manufacturer's protocol and sequenced on a NextSeq 550 system (Illumina, San Diego, CA) using the NextSeq® Reagent Kit v3 (150 cycles) with a PE NextSeq® Flow Cell. Data analysis was performed using the Illumina BaseSpace apps: TopHat Alignment (version 1.0.0, read mapping on the hg19 reference genome by TopHat21), fusion calling by TopHat-Fusion2, and RNA-seq Alignment (version 1.1.0, read mapping on the hg19 reference genome by STAR3, fusion calling by Manta4), using standard settings (https://basespace.illumina.com/apps). Fusion transcripts with a low number of split reads (<10) were excluded as likely false positives. Raw data of sequence variants were converted to variant call format (vcf) files and analyzed in Variant Studio software v.4.0.

**C Optical genome mapping (OGM)**

A calculated aliquot containing 1.5 million isolated mononuclear cells from bone marrow at diagnosis is used for the isolation of ultra-high molecular weight (UHMW) DNA, utilizing Nanobind paramagnetic disks following the manufacturer's protocol (Bionano Prep SP-G2 Frozen Cell Pellet DNA Isolation Protocol CG-00004-Rev-B). Subsequently, a fluorometric measurement of concentration and an evaluation of the homogeneity of the isolated DNA are performed using the Qubit dsDNA Assay Kit (Thermo Fisher Scientific). Labeling and staining are conducted according to the manufacturer's protocol (CG-30553-1 Bionano Prep® DLS-G2 Protocol). For labeling, 750 ng of UHMW DNA is utilized and labeled with the enzyme DLE-1, which recognizes the nucleotide sequence CTTAAG, in conjunction with the DLE-Green dye. DNA is stained using a mixture containing DTT and a DNA stain in the subsequent step. Following labeling and staining, a second fluorometric measurement and an assessment of homogeneity are conducted using the Qubit dsDNA HS Assay Kit (Thermo Fisher Scientific). The labeled genomic DNA (gDNA) is then loaded onto Saphyr G3.3 chips, and the molecules are visualized by the Saphyr instrument, which collects 2000 Gb of data per sample and is controlled by the Instrument Controller System (ICS) to optimize settings during data acquisition. Initially, analysis is performed using Bionano Access software, where the quality of the generated data is assessed. Following parameter assessment, the Rare Variants analysis is processed, with subsequent analysis utilizing the VIA software in a gene panel tailored for oncohematological samples (excluding MDS and AML) – PanHeme, using the recommended filters. Variant filtering involves the removal of changes with a low confidence ratio.

**D Whole-exome sequencing (WES)**

Whole-exome sequencing (WES) was performed using the Illumina DNA Prep with Exome 2.5 Enrichment kit (Illumina, San Diego, CA, USA) according to the manufacturer's protocol. Genomic DNA (approximately 500 ng) was used as input for library preparation. The libraries, normalized to a final concentration of 950 nM, were sequenced on the Illumina NextSeq 2000 platform using the S3 Reagent Kit (Illumina) in paired-end mode (2 × 150 bp), with a total insert size of approximately 300 bp. Raw sequencing reads were aligned to the GRCh37/hg19 human reference genome using the Burrows-Wheeler Aligner (BWA) algorithm. Variant calling was carried out using the Genome Analysis Toolkit (GATK). The resulting variant data were annotated and filtered using Variant Studio v3.0 (Illumina), and visualized using the Integrative Genomics Viewer (IGV) v2.4.

**List of cancer predisposition genes included in the analysis of germline variants**

| *A2ML1* | *CEP152* | *FAN1* | *KMT2C* | *NYNRIN* | *RB1* | *SMC3* |
| --- | --- | --- | --- | --- | --- | --- |
| *ABCB11* | *CEP164* | *FANCA* | *KMT2D* | *OFD1* | *RB1CC1* | *SMO* |
| *ABCB4* | *CEP57* | *FANCB* | *KRAS* | *OGG1* | *RBBP8* | *SMUG1* |
| *ABL1* | *CEP63* | *FANCC* | *LATS1* | *OPCML* | *RBM8A* | *SOCS2* |
| *ABL2* | *CETN2* | *FANCD2* | *LEF1* | *ORC1* | *RBSN* | *SOS1* |
| *ABRAXAS1* | *CHAF1A* | *FANCE* | *LIG1* | *P2RY12* | *RECK* | *SOS2* |
| *ACD* | *CHEK1* | *FANCF* | *LIG3* | *PALB2* | *RECQL* | *SPRED1* |
| *ADA* | *CHEK2* | *FANCG* | *LIG4* | *PARN* | *RECQL4* | *SPRTN* |
| *ADA2* | *CHIC2* | *FANCI* | *LMO1* | *PARP1* | *RECQL5* | *SRC* |
| *ADAMTS13* | *CIC* | *FANCL* | *LMO2* | *PARP2* | *REL* | *SRGAP1* |
| *AIP* | *CLCN5* | *FANCL* | *LPP* | *PARP3* | *RELA* | *SRGAP2* |
| *AK1* | *CLK2* | *FANCM* | *LRBA* | *PAX3* | *RELN* | *SRP54* |
| *AKT1* | *CLPB* | *FAS* | *LRP2* | *PAX5* | *REST* | *SRP72* |
| *AKT3* | *CLRN1* | *FASLG* | *LRP5* | *PAX6* | *RET* | *SRSF2* |
| *ALDH7A1* | *CMM* | *FBXW7* | *LUC7L2* | *PAX7* | *REV1* | *SRY* |
| *ALK* | *CNOT3* | *FCGR2A* | *LYST* | *PCNA* | *REV3L* | *SS18* |
| *ALKBH1* | *COL7A1* | *FCGR3B* | *LZTR1* | *PCNT* | *RFWD3* | *SSBP2* |
| *ALKBH2* | *CRB2* | *FEN1* | *MAD1L1* | *PDGFB* | *RHAG* | *STAG2* |
| *ALKBH3* | *CREBBP* | *FERMT1* | *MAD2L2* | *PDGFRA* | *RHBDF2* | *STAM* |
| *ALOX12B* | *CRIPAK* | *FERMT3* | *MAGT1* | *PDGFRB* | *RIF1* | *STAT1* |
| *AMELX* | *CRLF2* | *FGD3* | *MAML2* | *PDGFRL* | *RIT1* | *STAT2* |
| *AMELY* | *CSF1R* | *FGFR1* | *MAP2K1* | *PDPK1* | *RMI1* | *STAT3* |
| *ANK1* | *CSF3R* | *FGFR2* | *MAP2K2* | *PDS5b* | *RMI2* | *STAT4* |
| *ANKRD26* | *CTC1* | *FGFR3* | *MAX* | *PEAR1* | *RMRP* | *STAT5A* |
| *AP3B1* | *CTCF* | *FGTF2H4* | *MBD4* | *PF4* | *RNF168* | *STAT5B* |
| *APC* | *CTLA4* | *FH* | *MCC* | *PF4V1* | *RNF213* | *STAT6* |
| *APEX1* | *CTNNA1* | *FHL1* | *MDC1* | *PHB* | *RNF4* | *STK11* |
| *APEX2* | *CTNNB1* | *FLCN* | *MED12* | *PHF6* | *RNF6* | *STX11* |
| *APLF* | *CTR9* | *FLNA* | *MED12L* | *PHOX2A* | *RNF8* | *STXBP2* |
| *APTX* | *CUX1* | *FLT3* | *MEF2D* | *PHOX2B* | *ROCK2* | *SUFU* |
| *AR* | *CXCR2* | *FOSB* | *MEN1* | *PIAS1* | *ROR2* | *SUZ12* |
| *ARHGAP26* | *CXCR4* | *FOXP1* | *MET* | *PIAS2* | *ROS1* | *TAL1* |
| *ARHGEF12* | *CYCS* | *FOXP3* | *MGMT* | *PIAS3* | *RPA1* | *TBXA2R* |
| *ARID1A* | *CYLD* | *FPR1* | *MINPP1* | *PIAS4* | *RPA2* | *TCF3* |
| *ARID2* | *DCC* | *G6PC3* | *MITF* | *PICALM* | *RPA3* | *TCF7L2* |
| *ASXL1* | *DCLRE1A* | *G6PD* | *MLH1* | *PIK3CA* | *RPA4* | *TCIRG1* |
| *ATM* | *DCLRE1B* | *GALNT12* | *MLH3* | *PIK3CD* | *RPL10* | *TDG* |
| *ATP11C* | *DCLRE1C* | *GATA1* | *MLLT10* | *PIK3CG* | *RPL11* | *TERC* |
| *ATP7B* | *DDB1* | *GATA2* | *MLLT10* | *PIK3R1* | *RPL15* | *TERT* |
| *ATR* | *DDB2* | *GATA3* | *MLLT3* | *PIM1* | *RPL18* | *TET2* |
| *ATRIP* | *DDHD2* | *GBA* | *MMS19* | *PKHD1* | *RPL19* | *TFE3* |
| *ATRX* | *DDR2* | *GCLC* | *MN1* | *PLA2G2A* | *RPL23* | *TFRC* |
| *AURKA* | *DDX3X* | *GDNF* | *MNX1* | *PLAG1* | *RPL26* | *TGFBR1* |
| *AUTS2* | *DDX41* | *GFI1* | *MPL* | *PMS1* | *RPL27* | *TGFBR2* |
| *AXIN1* | *DIAPH1* | *GINS1* | *MPLKIP* | *PMS2* | *RPL31* | *TGFBR3* |
| *AXIN2* | *DIAPH2* | *GJB2* | *MPO* | *PNKP* | *RPL35* | *THPO* |
| *BAP1* | *DIAPH3* | *GNA11* | *MRE11* | *POLB* | *RPL35A* | *TINF2* |
| *BARD1* | *DICER1* | *GNAQ* | *MRE11A* | *POLD1* | *RPL36* | *TJP2* |
| *BAX* | *DIRC3* | *GNB1* | *MRTFA* | *POLE* | *RPL4* | *TLR2* |
| *BCC1* | *DIS3L2* | *GPC3* | *MSH2* | *POLH* | *RPL5* | *TMEM127* |
| *BCL10* | *DKC1* | *GRB2* | *MSH3* | *POLI* | *RPL9* | *TNFRSF14* |
| *BCL10* | *DLC1* | *GREM1* | *MSH4* | *POLK* | *RPS10* | *TNFRSF6* |
| *BCL11A* | *DLST* | *GTF2H1* | *MSH5* | *POLL* | *RPS15* | *TOPBP1* |
| *BCL11B* | *DMC1* | *GTF2H2* | *MSH6* | *POLM* | *RPS15A* | *TP53* |
| *BCL2* | *DNAJC21* | *GTF2H3* | *MTAP* | *POLN* | *RPS17* | *TP63* |
| *BCL3* | *DNM2* | *GTF2H4* | *MTNR1B* | *POLQ* | *RPS19* | *TPI1* |
| *BCL6* | *DNMT3A* | *GTF2H5* | *MTOR* | *POT1* | *RPS20* | *TPMT* |
| *BCL7A* | *DOCK8* | *H2AX* | *MUC2* | *POU6F2* | *RPS24* | *TREX1* |
| *BCL9* | *DROSHA* | *H3F3A* | *MUS81* | *PPBP* | *RPS26* | *TREX2* |
| *BCOR* | *DTNBP1* | *HAVCR2* | *MUTYH* | *PPM1D* | *RPS27* | *TRIM28* |
| *BCORL1* | *DUSP22* | *HAX1* | *MVK* | *PRF1* | *RPS27A* | *TRIM37* |
| *BCR* | *DUT* | *HBA1* | *MXI1* | *PRKAR1A* | *RPS28* | *TRIP13* |
| *BLM* | *EBF1* | *HBA2* | *MYB* | *PRKCB* | *RPS29* | *TRPM7* |
| *BLOC1S3* | *ECT2L* | *HBB* | *MYBL1* | *PRKCD* | *RPS7* | *TSC1* |
| *BMP4* | *EED* | *HEATR3* | *MYC* | *PRKDC* | *RPSA* | *TSC2* |
| *BMPR1A* | *EFL1* | *HELQ* | *MYCBP2* | *PRKN* | *RRAS* | *TSR2* |
| *BPGM* | *EGFR* | *HFE* | *MYCN* | *PROM1* | *RSPO1* | *TUBB1* |
| *BRAF* | *EGLN1* | *HGD* | *MYD88* | *PRPF19* | *RTEL1* | *TYK2* |
| *BRCA1* | *EIF2AK3* | *HLTF* | *MYH9* | *PRPF8* | *RUNX1* | *U2AF1* |
| *BRCA2* | *ELANE* | *HMBS* | *MYLK2* | *PRSS1* | *RYR2* | *U2AF2* |
| *BRIP1* | *EME1* | *HMCN1* | *NABP2* | *PTCH1* | *SAMD9* | *UBE2T* |
| *BTG1* | *EME2* | *HMGA2* | *NAF1* | *PTCH2* | *SAMD9L* | *UBE2V2* |
| *BTK* | *EP300* | *HMMR* | *NBEAL2* | *PTEN* | *SBDS* | *UNC13D* |
| *BUB1* | *EPAS1* | *HNF1A* | *NBN* | *PTGS1* | *SDHA* | *UROD* |
| *BUB1B* | *EPB41* | *HOXB13* | *NCOA2* | *PTPN11* | *SDHAF2* | *USH2A* |
| *BUB1B* | *EPB42* | *HPS1* | *NCOR1* | *PTPN12* | *SDHB* | *USP7* |
| *BUB3* | *EPCAM* | *HPS4* | *NCOR2* | *PTPN2* | *SDHC* | *UVSSA* |
| *C9* | *EPHA2* | *HRAS* | *NF1* | *PTPN6* | *SDHD* | *VHL* |
| *CALR* | *EPHB2* | *HUS1* | *NF2* | *PTPRC* | *SERPINA1* | *VPS13B* |
| *CASP10* | *EPO* | *HYOU1* | *NFIX* | *PTPRD* | *SETBP1* | *VPS45* |
| *CASP8* | *EPOR* | *IDH1* | *NHEJ1* | *PTPRJ* | *SETD1B* | *VWF* |
| *CASP8AP2* | *ERBB2* | *IDH2* | *NHP2* | *PTPRT* | *SETD2* | *WAS* |
| *CBFB* | *ERBB3* | *IGF2R* | *NIPBL* | *RAB27A* | *SETMAR* | *WRN* |
| *CBL* | *ERBB4* | *IKZF1* | *NOP10* | *RABGGTA* | *SF1* | *WT1* |
| *CCND1* | *ERCC1* | *IKZF3* | *NOS3* | *RAC1* | *SF3A1* | *WWOX* |
| *CCND2* | *ERCC2* | *IL7R* | *NOTCH1* | *RAC2* | *SF3B1* | *XAB2* |
| *CCND3* | *ERCC3* | *IRF1* | *NOTCH2* | *RAD17* | *SGK1* | *XIAP* |
| *CCNH* | *ERCC4* | *ITK* | *NOTCH3* | *RAD18* | *SH2B1* | *XPA* |
| *CD27* | *ERCC5* | *JAGN1* | *NOTCH4* | *RAD21* | *SH2B3* | *XPC* |
| *CD274(PD-L1)* | *ERCC6* | *JAK1* | *NPHP3* | *RAD23A* | *SH2D1A* | *XRCC1* |
| *CD36* | *ERCC8* | *JAK2* | *NPM1* | *RAD23B* | *SH3GL1* | *XRCC2* |
| *CD40LG* | *ERG* | *JAK3* | *NQO2* | *RAD50* | *SHC1* | *XRCC2* |
| *CD79B* | *ESR1* | *JMJD1C* | *NR4A3* | *RAD51* | *SHOC2* | *XRCC3* |
| *CD96* | *ETNK1* | *KAT6A* | *NRAS* | *RAD51A* | *SHPRH* | *XRCC4* |
| *CDC73* | *ETS1* | *KCNQ1OT1* | *NRG3* | *RAD51B* | *SLC22A18* | *XRCC5* |
| *CDC73* | *ETV6* | *KDM3B* | *NSD1* | *RAD51C* | *SLC25A13* | *XRCC6* |
| *CDH1* | *EWSR1* | *KDM3B* | *NSD2* | *RAD51D* | *SLX1B* | *ZBTB33* |
| *CDHR1* | *EXO1* | *KDM6A* | *NT5C2* | *RAD51D* | *SLX4* | *ZBTB7A* |
| *CDK4* | *EXT1* | *KDM6B* | *NTHL1* | *RAD52* | *SMAD4* | *ZFHX3* |
| *CDK7* | *EXT2* | *KDR* | *NTRK1* | *RAD54B* | *SMAD7* | *ZNF384* |
| *CDKN1B* | *EYS* | *KIF1B* | *NTRK2* | *RAD54L* | *SMARCA2* | *ZNF91* |
| *CDKN1C* | *EZH2* | *KIF1Bβ* | *NTRK3* | *RAD9A* | *SMARCA4* | *ZRSR2* |
| *CDKN2A* | *F8* | *KIT* | *NUDT1* | *RAF1* | *SMARCAL1* |  |
| *CDKN2B* | *F9* | *KLF6* | *NUP214* | *RAG1* | *SMARCB1* |  |
| *CEBPA* | *FAAP20* | *KLHDC8B* | *NUP98* | *RAG2* | *SMARCD2* |  |
| *CENPJ* | *FAAP24* | *KMT2A* | *NUTM1* | *RANBP17* | *SMARCE1* |  |
| *CENPP* | *FAH* | *KMT2B* | *NUTM2B-AS1* | *RARA* | *SMC1A* |  |

**E Sanger sequencing**

Direct DNA sequencing using the Sanger method was performed to validate sequence changes in the *PAX5* gene. Gene-specific primers were designed to amplify the regions of interest. PCR amplification was carried out using Q5® High-Fidelity DNA Polymerase (New England Biolabs), which possesses 3′→5′ exonuclease (proofreading) activity. PCR products were purified using the CleanUp Concentrator Kit. Sequencing reactions were conducted using the BigDye™ Terminator v3.1 Cycle Sequencing Kit (Applied Biosystems), followed by purification with the BigDye XTerminator™ Purification Kit (Applied Biosystems). Capillary electrophoresis was performed using the 3500Dx Genetic Analyzer (Applied Biosystems). Final sequence data were analyzed using Sequencher v5.0 software.

**S6 Review of the molecular and clinical characteristics of the described *PAX5* germline variants**

| AA residue | Domain | DNA notation | Protein notation | Phenotype | Penetrance | No. of subjects/families (country) | Year/References |
| --- | --- | --- | --- | --- | --- | --- | --- |
| 38 | N-terminal paired domain | c.113G>A | p.Arg38His | BCP-ALL / healthy carriers | 1/2 (50.0%) | 2/1 (Iran) | 2015 / Yazdanparast (2) |
| 38 | N-terminal paired domain | c.113G>A | p.Arg38His | BCP-ALL / healthy carriers | 3/4 (75.0%) | 4/1 (France) | 2021 / Duployez (3) |
| 99 | N-terminal paired domain | c.295dup | p.Ile99Asnfs*3 | BCP-ALL / healthy carriers | 1/3 (33.3%) | 3/1 (Poland) | 2025 / Urbański |
| 183 | octapeptide domain | c.547G>A | p.Gly183Ser | BCP-ALL / healthy carriers | 3/8 (37.5%) | 8/1 (Germany, Israel) | 2013 / Auer (4) |
| 183 | octapeptide domain | c.547G>A | p.Gly183Ser | BCP-ALL / healthy carriers | 8/11 (72.7%) | 11/2 (USA, Puerto Rico) | 2013 / Shah (5) |
| 183 | octapeptide domain | c.547G>A | p.Gly183Ser | BCP-ALL / healthy carriers | 4/8 (50.0%) | 8/1 (Ecuador, Japan) | 2022 / Escudero (6) |
| 183 | octapeptide domain | c.548delG | p.Gly183Alafs*84 | BCP-ALL / healthy carriers | 2/4 (50.0%) | 4/1 (Italy) | 2024 / Bettini (7) |
| 322 | C-terminal transactivation domain | c.963del | p.Ala322Leufs*11 | BCP-ALL / healthy carriers | 2/2 (100.0%) | 2/1 (Mexico) | 2024 / García Solorio (8) |
| 338 | C-terminal transactivation domain | c.1013-2A>G | N/A | BCP-ALL | 1/1 (100.0%)* | 1/1 (UK) | 2015 / Stasevich (9) |
| N/A | N/A | 9p13.2(36960765_36975108)x1 | deletion resulting in a truncated protein | BCP-ALL | 1/1 (100.0%)* | 1/1 (The Netherlands) | 2023 / van Engelen (10) |

Abbreviations: AA – amino acid, BCP – B-cell precursor acute lymphoblastic leukemia
* proband’s family not verified

**References**

1. Pia̧tosa B, Wolska-Kuśnierz B, Pac M, Siewiera K, Gałkowska E, Bernatowska E. B cell subsets in healthy children: Reference values for evaluation of B cell maturation process in peripheral blood. Cytom Part B - Clin Cytom. 2010 Nov;78(6 B):372–81.

2. Yazdanparast S, Khatami SR, Galehdari H, Jaseb K. One missense mutation in exon 2 of the PAX5 gene in Iran. Genet Mol Res. 2015 Dec 22;14(4):17768–75.

3. Duployez N, Jamrog LA, Fregona V, Hamelle C, Fenwarth L, Lejeune S, et al. Germline PAX5 mutation predisposes to familial B-cell precursor acute lymphoblastic leukemia. Blood. 2021 Mar 11;137(10):1424–8.

4. Auer F, Rüschendorf F, Gombert M, Husemann P, Ginzel S, Izraeli S, et al. Inherited susceptibility to pre B-ALL caused by germline transmission of PAX5 c.547G>A. Leukemia. 2013 Nov 29;28(5):1136–8.

5. Shah S, Schrader KA, Waanders E, Timms AE, Vijai J, Miething C, et al. A recurrent germline PAX5 mutation confers susceptibility to pre-B cell acute lymphoblastic leukemia. Nat Genet. 2013 Sep 8;45(10):1226–31.

6. Escudero A, Takagi M, Auer F, Friedrich UA, Miyamoto S, Ogawa A, et al. Clinical and immunophenotypic characteristics of familial leukemia predisposition caused by PAX5 germline variants. Leukemia. 2022 Sep 1;36(9):2338–42.

7. Bettini LR, Fazio G, Saitta C, Piazza R, Palamini S, Buracchi C, et al. Diverse mechanisms of leukemogenesis associated with PAX5 germline mutation. Leukemia. 2024 Nov 1;38(11):2479–82.

8. García-Solorio J, Martínez-Villegas O, Rodríguez-Corona U, Molina-Garay C, Jiménez-Olivares M, Carrillo-Sanchez K, et al. Case report: A familial B-acute lymphoblastic leukemia associated with a new germline pathogenic variant in PAX5. The first report in Mexico. Front Oncol. 2024 Mar 20;14:1355335.

9. Stasevich I, Inglott S, Austin N, Chatters S, Chalker J, Addy D, et al. PAX5 alterations in genetically unclassified childhood Precursor B-cell acute lymphoblastic leukaemia. Br J Haematol. 2015 Oct 1;171(2):263–72.

10. van Engelen N, Roest M, van Dijk F, Sonneveld E, Bladergroen R, van Reijmersdal S V., et al. A novel germline PAX5 single exon deletion in a pediatric patient with precursor B-cell leukemia. Leukemia. 2023 Sep 1;37(9):1908–11.
